# Complete mitochondrial genome of *Melanabropsis tianmuica* (Anabropsinae: Anostostomatidae) from China

**DOI:** 10.1128/mra.00612-24

**Published:** 2025-05-28

**Authors:** Siyu Pang, Yingying Zhang, Yanting Qin, Xun Bian

**Affiliations:** 1 Ministry of Education, Key Laboratory of Ecology of Rare and Endangered Species and Environmental Protection, Guangxi Normal University 12388, Guilin, China; 2 Guangxi Key Laboratory of Rare and Endangered Animal Ecology, Guangxi Normal University 12388, Guilin, China; University of Maryland School of Medicine, Baltimore, Maryland, USA

**Keywords:** anabropsinae, *Melanabropsis*, mitogenomes

## Abstract

The *Melanabropsis tianmuica* mitogenome was 15,696 bp in length and consisted of 13 protein-coding genes, 22 transfer RNA genes, and 2 ribosomal RNA genes. All 13 protein-coding genes started with the ATN codons and terminated with TAA/TAG, except *cox2* and *nad5*, which ended with incomplete T.

## ANNOUNCEMENT

*Melanabropsis* was a newly erected genus by Wang and Liu, comprising four Chinese species and three Japanese species (1, 2). Currently, there are only morphological descriptions available for *Melanabropsis*, with no molecular data sequenced yet (2). The release of the mitochondrial genome for *Melanabropsis tianmuica* will aid in our future phylogenetic studies. *M. tianmuica* has a black body and is wingless, and it tends to be active during the night, seeking food or engaging in other activities.

In this study, the sample of *M. tianmuica* was collected from Qingliangfeng, Zhejiang, China (30.1051 N, 118.8893 E) and was stored in 100% ethanol at −4°C. The specimen was deposited in Guangxi Normal University (Guangxi, China; Xun Bian, xunbian2010@163.com). Total genomic DNA was extracted from the muscle of hind femur using the TIANamp Genomic DNA Kit (TIANGEN) following the instructions and sent to Beijing Berry Genomics Co., Ltd. for high-throughput sequencing. The 150-base-pair paired-end library was constructed with the MGIEasy Kit (MGI) as well as sequenced on an Illumina Hiseq 2500 (Illumina Inc.). The raw data were processed with fastp v.0.20.0 (3), by trimming adapters and primers, filtering reads with phred quality <Q5 and filtering reads with N base number >3. The sequencing generated 5,938,596,900 reads that were filtered. The number of reads listed is after QC. The raw paired-end reads were filtered to obtain high-quality clean reads by using CLC Genomics Workbench 12 with default parameters (4). The mitochondrial genome of *Henicus brevimucronatus* (NC_028063) served as the reference sequence, and the new mitochondrial genome was assembled with NOVOPlasty 4.2 (5), employing type mito, genome range of 14,000–18,000, kmer 39, yielding an N50 of 15,696. Annotation was performed using the MITOS2 tool available on Galaxy (https://usegalaxy.org) (6), utilizing the Invertebrate genetic code and RefSeq 89 Metazoa reference data. The mitogenome maps were obtained using the CGView server online tool (https://proksee.ca/) (7).

**Fig 1:**
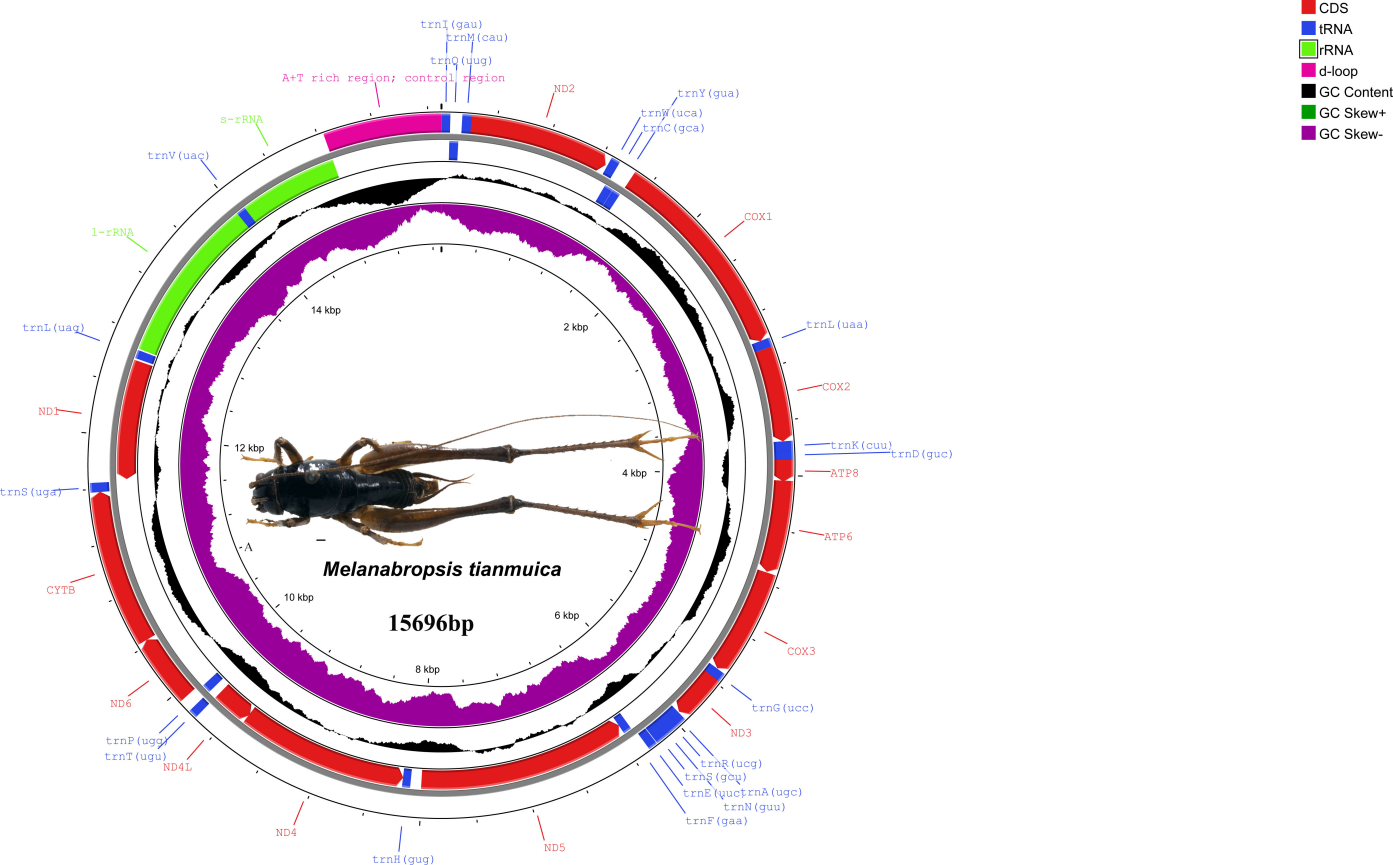
Mitogenome map of the species of *M. tianmuica*. The genomes transcribed clockwise are on the outermost circle, while counterclockwise transcribed anticlockwise are in the second circle. The color coding corresponds to genes of different groups as listed in the key in the upper right. PCG, protein-coding genes.

The newly sequenced mitochondrial genome of *M. tianmuica* measures 15,696 bp and exhibits an AT bias of 67.1% (GenBank accession number PP764156) (Fig. 1). The GC content is 32.9%. This mitogenome features a typical arthropod gene arrangement and includes 13 protein-coding genes, 22 transfer RNA genes, 2 ribosomal RNA genes, and a control region. Comparative analysis showed that the A+T content was higher than the C+T content in whole mitogenomes (Table 1). All 13 protein-coding genes initiated with the typical ATN codons and terminated with complete TAA/TAG, except cox2 and nad5, which ended with an incomplete T, a common occurrence in animal mitochondrial genomes (8, 9). The most frequently used amino acids of 13 protein-coding genes were UUA (Leu2) and UCA (Ser2).

**TABLE 1:** Nucleotide composition of different regions of the mitogenomes

Species	Whole mitochondrial genome			tRNAs			rRNAs			PCGs			PCGs-1			PCGs-2			PCGs-3		
	A+T (%)	AT skew	GC skew	A+T (%)	AT skew	GC skew	A+T (%)	AT skew	GC skew	A+T (%)	AT skew	GC skew	A+T (%)	AT skew	GC skew	A+T (%)	AT skew	GC skew	A+T (%)	AT skew	GC skew
*Melanabropsis tianmuica*	67.1	0.068	−0.349	72.2	0.001	0.146	68.7	−0.081	0.416	65.3	−0.148	−0.078	60.6	−0.04	0.122	63.7	−0.397	−0.153	71.5	−0.019	−0.26

## Data Availability

The genome sequence data that support the findings of this study are openly available in NCBI GenBank at https://www.ncbi.nlm.nih.gov/nuccore/PP764156 under the reference number PP764156. Species numbers are determined on the Orthoptera Species File available from: https:// Orthoptera.SpeciesFile.org. The software used in data assembly, CLC Genomics Workbench 12, NOVOPlasty 4.2, MITOS2 of Galaxy, and CGView is available for download or use from official channels. The associated BioProject, SRA, and BioSample numbers are PRJNA1177765, SRR31126561, and SAMN44443355, respectively.
